# What Becomes of Some Spoiled Drugs

**Published:** 1899-04

**Authors:** 


WHAT BECOMES OF SOME SPOILED DRUGS. A recent writer in a drug journal, speaking of the best manner of disposing of crude drugs that have deteriorated in quality through age or other causes, says: "One dislikes to throw them away, as he should properly do. Whenever I discover that any roots, barks, berries, etc., either whole or in powder, have begun to deteriorate I immediately replace them with fresh goods and reserve the spoiled articles for the next batch of condition-powders." To the veterinarian relying upon such a person for pure and reliable drugs the moral is obvious.

The bite of the tsetse fly, so deadly to the horse, ox, and dog, has been found by a committee of the British Royal Society to affect other creatures as well. No remedy has been found. This little insect is playing a surprising part in the world's development, as, although man is strangely immune to the poison, large districts in Africa—notably the Limpopo and Zambesi Valleys—must remain uninhabitable until the pest shall have been destroyed.

---

The new corn product upon which investigations have been conducted at the Maryland Agricultural Experiment Station, to determine its value as a stock food, has been given much consideration by the farming community of the State; in fact, many States have taken up the subject and are conducting experiments to ascertain its relative feeding value. In the process of the extraction of the pith the blades and husks are first removed and the stalks are cut up in small pieces. After the extraction of the pith from the stalk the balance is ground up into meal, which in general appearance resembles coarse bran. This ground material is termed the "new corn product." The new corn product contains eleven pounds per hundred more digestible matter and two pounds per hundred more digestible protein than the whole fodder shredded. During the last few years much has been done in the way of testing methods for preparing corn-fodder for feeding, with most of the results in favor of some method of shredding the stalk. Shredding possesses many points which make it superior to the ordinary or old way of cutting fodder, the principal one of which is that the shredded fodder is almost wholly eaten by animals.

It is found that the new corn product contains within one pound as much total digestible matter as wheat-bran, but less than one-third as much digestible protein; consequently the nutritive ratio is wider. It was further observed that animals fed with a fattening ration with the new corn product base made more gain in live weight and upon less feed than with a fattening ration of the same grain and corn-blades. The keeping qualities of the new corn product are as good as linseed meal, cottonseed meal, or wheat-bran, and rations made up with this material can be fed with less labor and less waste of feed than when hay and fodder are fed separately as ordinarily practised.—*Baltimore Sun.*

